# Prevention Practice of COVID-19 Using Personal Protective Equipment and Hand Hygiene Among Healthcare Workers in Public Hospitals of South Wollo Zone, Ethiopia

**DOI:** 10.3389/fpubh.2021.782705

**Published:** 2021-12-02

**Authors:** Awoke Keleb, Ayechew Ademas, Mistir Lingerew, Tadesse Sisay, Gete Berihun, Metadel Adane

**Affiliations:** Department of Environmental Health, College of Medicine and Health Science, Wollo University, Dessie, Ethiopia

**Keywords:** COVID-19, compliance, hand hygiene, health care, personal protective equipment

## Abstract

**Objective:** The use of personal protective equipment and hand hygiene are often the most recommended line of defense against coronavirus disease-19 (COVID-19). The purpose of this study is to determine the magnitude of compliance and associated factors of personal protective equipment (PPE) utilization and hand hygiene practice among healthcare workers in public hospitals of South Wollo Zone, Northeastern Ethiopia.

**Methods:** A hospital-based cross-sectional study was conducted among 489 healthcare workers in public hospitals of South Wollo Zone, Northeastern Ethiopia from June 15 to July 30, 2021. Proportional sample size allocation to each selected hospital followed by simple random sampling techniques were used to select the study participants using human resource records from each hospital. A pre-tested and structured self-administered questionnaire with WHO's standardized hand hygiene and PPE utilization observational checklist were used to collect data. Bivariate and multivariable analyses with 95% CI and *p*-value < 0.05 were employed to identify the associated factors of personal protective equipment utilization.

**Results:** About 32 and 22.3% of healthcare workers were compliant with personal protective equipment utilization and hand hygiene practice, respectively. Feedback for safety (AOR = 2.05; 95% CI: 1.26–3.35), training on COVID-19 prevention (AOR = 3.43; 95% CI: 2.01–5.86), and perception to infection risk (AOR = 1.98; 95% CI: 1.18–3.33) were significant factors of good compliance with personal protective equipment utilization.

**Conclusion:** The magnitude of good compliance with personal protective equipment utilization and hand hygiene was low. Interventions to promote personal protective equipment utilization and hand hygiene should focus on feedback for safety, training on COVID-19 prevention, and perception of infection risk.

## Introduction

The coronavirus (COVID-19) pandemic has overwhelmingly changed the world and, consequently, is changing the conditions of healthcare workers (HCWs) (1). This pandemic is creating profound challenges for healthcare workers and healthcare systems in the world, as the disease is spreading at an alarming rate, surpassing hospital capacities and exposing healthcare workers to a high risk of exposure (2). The outbreak of severe acute respiratory syndrome coronavirus (SARS-CoV-2) was first reported in Wuhan, Hubei province, China, in late December 2019 and has rapidly spread to other countries (1).

SARS-CoV-2 is especially transmitted through droplets and touch (3) especially during airway maneuvers in an infected patient, like during tracheal intubation (4, 5). The majority of people infected with the coronavirus are associated with occupational exposure. COVID-19 could also be the primary new industrial disease during this decade (6). It is believed that the primary occupational groups in danger are persons working in seafood and wet animal markets in Wuhan (3).

As of July 2021, over 206 million confirmed cases of COVID-19, the disease caused by SAR-CoV-2, and close to 4.4 million confirmed deaths were reported by the World Health Organization (WHO) (4). The cumulative number of cases within the African continent is over 6.5 million (6,587,734) confirmed COVID-19 cases which accounts for 3.4% of the total cases reported globally, and 167,183 deaths with a 2.5% fatality rate (4, 5). A global systematic review indicated that a total of 152,888 infections and 1,413 deaths were reported among healthcare workers worldwide. Infections were mainly in women and nurses but deaths were mainly in men and doctors (7).

The people most in danger of infection are those that are in close contact with a COVID-19 patient or who look after COVID-19 patients. Subsequently, healthcare workers are a subsequent high-risk group to accumulate this infection (8). According to OSHA, high-risk workers include those involved in healthcare, death care, laboratories, airline operations, solid waste, and wastewater management and visit areas where the virus is spreading (9).

Since, HCWs are putting themselves at high risk of COVID-19, measures to stop SARS-CoV-2 transmission in healthcare staff are an instantaneous priority (5, 10); therefore, HCWs are required to protect themselves and stop transmission within the healthcare setting (3) since the health and well-being of our healthcare workers determine our nation's health, security, and economic prosperity.

Of concern, doctors are significantly suffering from COVID-19 in Africa, with 14,148 HCWs being infected in many counties since the start of the outbreak. Overall, South Africa has been the foremost affected, with 4,842 (34%) infected, followed by Algeria (2,300), Ghana (2,065), Nigeria (987), Cameroon (593), Senegal (271), Guinea-Bissau (268), Malawi (264), Guinea (244), Côte d'Ivoire (187), Liberia (184), Niger (184), Sierra Leone (168), and Ethiopia (87) (2, 11).

WHO and other national and international public health authorities recommend proper personal protective equipment (PPE) utilization and hand hygiene compliance (3, 5). As a result, any potential transmission can be prevented, thereby HCWs are often protected. Although the foremost effective interventions to protect HCWs are to physically separate HCWs from infectious patients and body fluids, mortality rates of COVID-19-infected patients are often decreased with more aggressive care that needs close contact with these patients (12, 13).

During this setting, adhering to PPE utilization and hand hygiene practice are the last physical barrier between a healthcare provider and infectious body fluids (2, 6). However, there is a big discrepancy concerning access and utilization of PPE and hand hygiene protocols which are not always followed in many medical institutions during COVID-19 patient management.

The speed with which COVID-19 is spreading across the world involves an assessment of the reality of healthcare workers' PPE utilization and hand hygiene during the COVID-19 pandemic (1, 3, 14). Even though hand hygiene is the most critical intervention for protecting HCWs from infections including COVID-19, the compliance rate for hand hygiene has not drastically improved (15). This study aims to determine compliance of personal protective equipment utilization and hand hygiene practice and associated factors among healthcare workers toward the COVID-19 pandemic in hospital settings.

## Methods

### Study Setting

**Figure 1:**
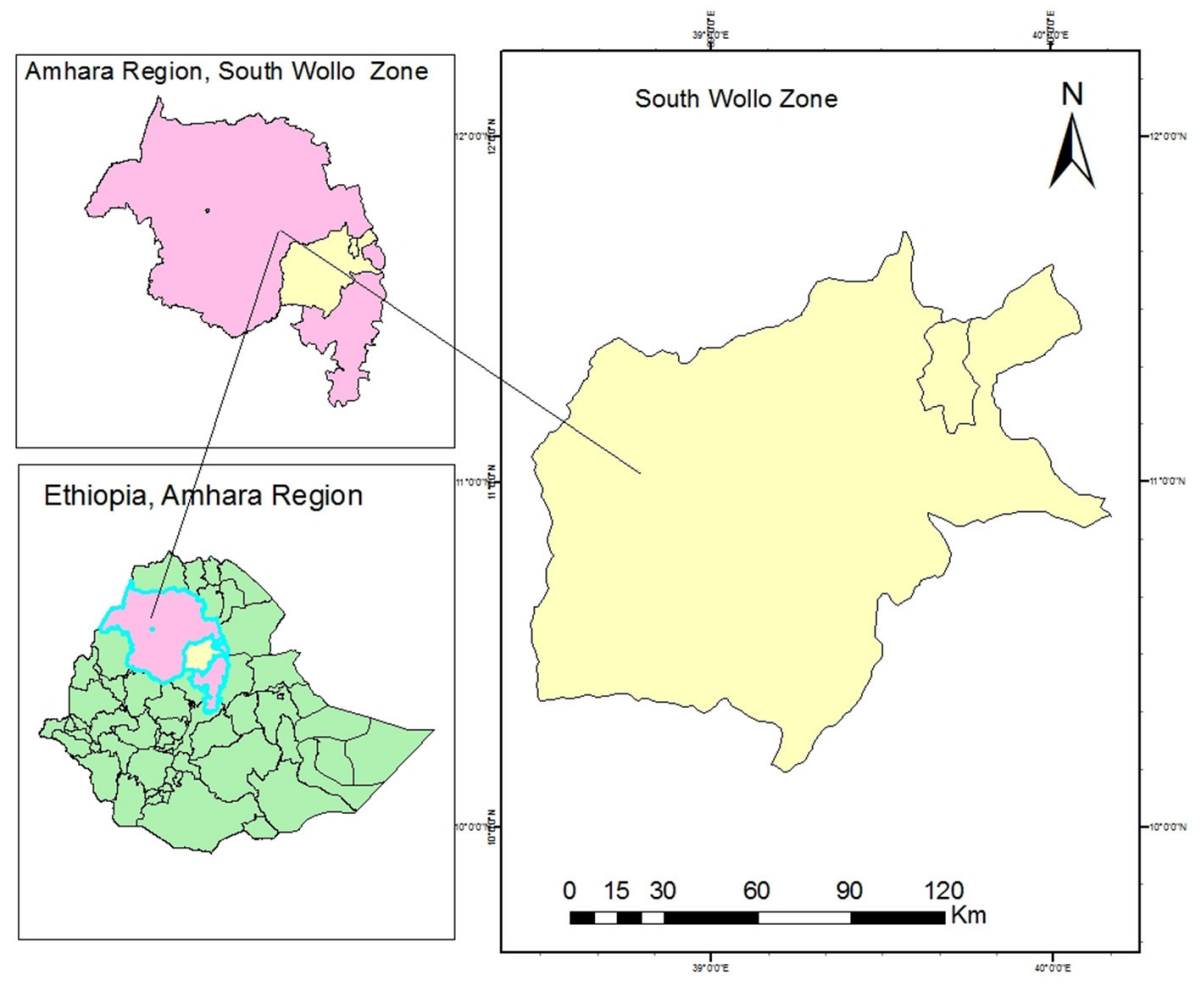
Map of the study area.

South Wollo Zone is one among 10 zones within the Amhara Region of Ethiopia (Figure 1).

Based on the 2007 Census conducted by the Central Statistical Agency of Ethiopia (CSA), this zone features a complete population of 2,518,862, an increase of 18.60% over the 1994 census, of whom 1,248,698 are men and 1,270,164 are women in an area of 17,067.45 square kilometers. South Wollo has a population density of 147.58. While 301,638 (11.98%) are urban inhabitants, a further 2,217,224 (88.02%) inhabitants were reported to be rural. A total of 598,447 households were counted in this zone, which can be calculated in a mean of 4.21 persons to a household, and 574,378 housing units. There are seven public hospitals in the South Wollo Zone with a total of 1,051 healthcare workers to serve the catchment population of the South Wollo Zone and the nearby zones especially for the Afar region.

### Study Design and Period

A hospital-based cross-sectional study was conducted to assess the magnitude of compliance with personal protective equipment utilization and hand hygiene and its associated factors among healthcare workers in public hospitals of South Wollo Zone, Northeastern Ethiopia from June 15 to July 30, 2021.

### Source Population and Inclusion Criteria

The source population of this study was all healthcare workers working in South Wollo Zone hospitals while the study population was all selected healthcare workers in South Wollo Zone hospitals. From the study population, all permanent healthcare workers employed in the hospitals were included in the study.

### Sample Size Determination and Sampling Procedure

The sample size was determined using the single population proportion formula with the following assumptions: Magnitude of compliance with standard precaution practice (*p* = 12%) was taken from a study conducted in Gondar University Comprehensive Specialized Hospital, Northwest Ethiopia (16).

$n=\frac{{\left(Z_{a/2}\right)}^{2}\;*\;p\left(1-p\right)}{d^{2}}$ where **n** is the optimum sample size required**, P** is an estimate of the magnitude of compliance with standard precaution**, Z** is the standard normal variable at a (1-α) % confidence level, α is mostly 0.05, i.e., with 95% CI (*z* = 1.96), and **d** is the margin of error to be tolerated (%).

The determination of the margin of error is based on the optimum sample size and availability of resources considering one percent (1%) giving the largest sample size and 5% giving the smallest sample size. For this study, a margin of 3% was used which was based on the proportion of 12% taken from a similar study mentioned above, which gave an adequate sample size.

(1) $$\begin{array}{r} n=\frac{{\left(1.96\right)}^{2}*0.12\left(1-0.12\right)}{{\left(0.03\right)}^{2}}=451 \end{array}$$

After adding a 10% non-response rate the final sample size was ***n* = 496**.

There are seven public hospitals in the South Wollo Zone from which three hospitals were selected randomly. All 496 estimated participants were proportionally allocated to each hospital based upon their respective numbers of healthcare workers. The sample size from each hospital was proportionally allocated based on the types of profession (strata) and numbers of their healthcare workers. The study participants were selected using a simple random sampling method within their strata using human resource records from each hospital.

### Dependent and Independent Variables

#### Dependent Variables

- Personal protective equipment utilization (good compliance/poor compliance).
- Hand hygiene practice (good compliance/poor compliance).

#### Independent Variables

##### Socio-Demographic Factors

Age of respondent, sex, marital status, type of profession, educational status and work experience, and respondent working unit.

##### Institutional Factors

Availability of PPE, presence of COVID guidelines, and feedback for safety.

##### Behavioral Factors

Infection prevention training on COVID-19, perception of infection risk, drinking alcohol, chewing khat, and hand hygiene practice.

#### Operational Definition

##### PPE Utilization and Hand Hygiene Measurement

Compliance was measured using data from direct observations by trained BSc nurses.

##### Compliance of Personal Protective Equipment Utilization

Good compliance with PPE utilization was considered when the HCW scored more than or equal to the mean score, and a score less than the mean score was taken as “poor compliance” from the observational checklist (17–19).

##### Hand Hygiene Compliance

Good hand hygiene compliance was considered when the HCW scored more than or equal to the mean score from the observational checklist (20, 21).

### Data Collection Tools and Quality Assurance

The data were collected using WHO's standardized hand hygiene and PPE utilization observational checklist (22–24). The observation was focused on six moments of PPE use: wearing a face mask, eye goggle, apron, glove, and gown, while observation for hand hygiene was focused on six domains: washing hands before touching a patient, before clean or aseptic procedures, after body fluid exposure, after touching a patient, immediately after removal of gloves, and between patient contact. Before actual data collection, six observers (BSc nurses) and three supervisors (public health experts) were trained for two days in accordance with WHO hand hygiene and PPE utilization techniques focused on each item on the observational checklist plus additional time for observing the practice and considering ethical issues. After training, a pre-test was conducted on 25 healthcare workers in nearby Woldeya Hospital, North Wollo Zone, Ethiopia.

During observation, the data collectors directly observed the study participants while the HCWs conducted clinical examinations on patients. The observation was made on nine units in the selected hospitals: emergency room, pediatrics ward, delivery/gynecology ward, medical ward, surgical ward, operation theater unit, laboratory, radiology unit, recovery, outpatient department (OPD), and a physiotherapy room. Along with standing concern with clinical observation is the Hawthorne effect, in which study subjects' awareness of being observed causes them to alter their behavior. To minimize such bias, data collectors were coached to observe discretely in which the HCWs were unaware of the research activities. Some days after the completion of observation, a self-administered pretested structure questionnaire was distributed to the same HCWs to collect other required information such as socio-demographic, institutional, and behavioral factors. Three public health experts supervised the data collection process including observation and completeness of questionnaires by giving daily feedback to data collectors before data entry.

### Statistical Analysis

Data were entered using EpiData version 3.1 and exported to Statistical Package of Social Science (SPSS) version 25.0 for data cleaning and analysis. Once the data were entered, basic quality assurance measures including data cleaning using browsing of data tables after sorting, frequency distributions, and cross-tabulations and summary statistics using sorting were performed. Descriptive statistics were used for socio-demographic characteristics and mean ± SD (standard deviations) for continuous variables. Continuous variables were categorized using information from the literature, and categorical variables were re-categorized accordingly.

Bivariate (crude odds ratio [COR]) and multivariable (adjusted odds ratio [AOR]) values were calculated using logistic regression analysis with a 95% confidence interval [CI]. From the bivariate analysis, variables with *p* < 0.25 were considered as candidate variables for multivariable analysis and AOR was determined after adjusting for the availability of PPE, feedback for safety, training on COVID-19 prevention, perception to infection risk, drinking alcohol, and chewing khat using the backward stepwise method. From the multivariable logistic regression analysis, variables with a significance level of *p* < 0.05 were taken as statistically significant and independently associated with compliance with personal protective equipment utilization. The presence of multi-collinearity among independent variables was checked using standard error at the cutoff value of 2. Model fitness was checked using the Hosmer-Lemeshow test which had a *p*-value > 0.05.

## Results

### Socio-Demographic Characteristics of the Respondents

A total of 489 healthcare workers were observed and completed the survey with a response rate of 98.6%. More than half of the participants were women which accounts for 276 (56.4%), nearly half 238 (48.7%) of the HCWs were married, and the majority of the participants 292 (59.7%) were nurses. About two-thirds 343 (70.2%) of the respondents had Bachelor degrees and 367 (75.1%) of the respondents had >10 years of work experience (Table 1).

**Table 1:** Socio-demographic characteristics of healthcare workers in public hospitals of South Wollo Zone, Northeastern Ethiopia, 2021.

**Variables**	**Category**	**Frequency (n)**	**Percentage (%)**
Sex	Male	213	43.6
	Female	276	56.4
Age of respondent	19–30	156	41.9
	31–40	172	46.2
	41 and above	44	11.8
Marital status	Currently unmarried	251	51.3
	Currently married	238	48.7
Respondent working unit	Emergency room	33	6.7
	Pediatrics ward	45	9.2
	Delivery or gyn ward	81	16.6
	Medical ward	68	13.9
	Surgical ward	22	4.5
	Operation theater unit	42	8.6
	Laboratory	63	12.9
	Radiology unit	32	6.5
	Recovery	33	6.7
	OPD	53	10.8
	Physiotherapy room	17	3.5
Educational status	Certificate	70	14.3
	Diploma	76	15.5
	BSc	199	40.7
	Medical doctor	107	21.9
	MSc/specialist	37	7.6
Work experience in years	>10 years	367	75.1
	5–10 years	95	19.4
	<5 years	27	5.5
Types of profession	Nurses	292	59.7
	Medical doctor	122	24.9
	Laboratory	39	8.0
	Other allied HCWs	36	7.4

### Institutional and Behavioral Factors

More than three-quarters 381 (77.9%) of the healthcare workers had personal protective equipment in their working department and nearly three-quarters 361 (73.8%) reported having COVID-19 guidelines as a working protocol for COVID-19 management but less frequent feedback for safety 390 (79.8%) was given by the infection prevention officers. Even though, nearly two-thirds 300 (61.3%) of the HCWs had a perception of infection risk, only half 256 (52.4%) of healthcare workers had taken training on COVID-19 prevention and control (Table 2).

**Table 2:** Institutional and behavioral factors of healthcare workers in public hospitals of South Wollo Zone, Northeastern Ethiopia, 2021.

**Variable**	**Category**	**Frequency (n)**	**Percentage (%)**
Availability of PPE	No	108	22.1
	Yes	381	77.9
Presence of COVID guidelines	No	128	26.2
	Yes	361	73.8
Feedback for safety	Less frequent	390	79.8
	More frequent	99	20.2
Training on COVID-19	No	233	47.6
	Yes	256	52.4
Perception to infection risk	No	189	38.7
	Yes	300	61.3
Drinking alcohol	No	391	80.0
	Yes	98	20.0
Chewing khat	No	423	86.5
	Yes	66	13.5

### Personal Protective Equipment Utilization

In the routine care of patients in the healthcare setting, most HCWs reported that they always wear FFP2/N95 facemasks (245, 50.1%), gowns and gloves were used for routine care among 305 (62.4%) and 319 (65.2%) HCWs, respectively while 156 (31.9%) HCWs used eye goggles and aprons independently. According to this study, the overall number of HCWs who were compliant with personal protective equipment utilization was found to be 156 (31.9%) (95% CI: 27.9–36.6) (Table 3). Furthermore, the least compliant healthcare workers were laboratory professionals followed by nurses and doctors (Table 4).

**Table 3:** Compliance of PPE utilization measurement indications among healthcare workers in public hospitals of South Wollo Zone, Northeastern Ethiopia, 2021.

**PPE use indications**	**No**	**Yes**
Wearing facemasks	244 (49.9%)	245 (50.1%)
Wearing eye goggles	333 (68.1%)	156 (31.9%)
Wearing aprons	331 (67.7%)	158 (32.3%)
Wearing gloves	170 (34.8%)	319 (65.2%)
Wearing gowns	184 (37.6%)	305 (62.4%)
Overall PPE utilization compliance	Poor compliance	333 (68.1%)
	Good compliance	156 (31.9%)

**Table 4:** Proportion of HCW compliance with PPE utilization by professionals in public hospitals of South Wollo Zone, Northeastern Ethiopia, 2021.

**Professions**	**PPE utilization**			
	**Good compliance**		**Poor compliance**	
	**Frequency**	**Percentage**	**Frequency**	**Percentage**
Nurses	107	21.88%	185	37.83%
Doctors	28	5.73%	30	6.13%
Laboratory	9	1.84%	94	19.22%
Other allied HCWs	12	2.45%	24	4.92%
Total	156	31.9%	333	68.1%

### Hand Hygiene Measurement Domains

The potential range of cumulative hand hygiene domain score was 0–6, and we assessed a mean score of 3.56 ± 1.31. According to the observational result, only one-third 153 (31.3%), more than half 269 (55.0%), and much more than three-quarters 427 (87.3%) of them practiced hand washing before touching a patient, before clean or aseptic procedures, and after body fluid exposure, respectively. Moreover, 157 (32.1%), 429 (87.7%), and 176 (36.0%) HCWs practiced hand washing after touching a patient, immediately after removal of gloves, and between patient contact, respectively. The overall number of HCWs who had good compliance with hand hygiene was found to be 294 (22.3%) (95% CI: 18.8–26.0) (Table 5).

**Table 5:** Hand hygiene domains among healthcare workers toward COVID-19 in public hospitals of South Wollo Zone, Northeastern Ethiopia, 2021.

**Domains**	**No**	**Yes**
Washing hands before touching a patient	336 (68.7%)	153 (31.3%)
Washing hands before clean or aseptic procedures	220 (45.0%)	269 (55.0%)
Washing hands after body fluid exposure	62 (10.2%)	427 (87.3%)
Washing hands after touching a patient	332 (67.8%)	157 (32.1%)
Washing hands immediately after removal of gloves	60 (12.3%)	429 (87.7%)
Washing hands between patient contact	313 (64.0%)	176 (36.0%)
Overall hand hygiene compliance	Poor compliance	380 (77.7%)
	Good compliance	294 (22.3%)

### Factors Associated With Personal Protective Equipment Utilization

The adjusted logistic regression analysis result indicated that feedback for safety (AOR = 2.05; 95% CI: 1.26–3.35), training on COVID-19 prevention (AOR = 3.43; 95% CI: 2.01–5.86), and perception to infection risk (AOR = 1.98; 95% CI: 1.18–3.33) were significantly associated with compliance of personal protective equipment utilization (Table 6).

**Table 6:** Factors associated with personal protective equipment utilization among healthcare workers toward COVID-19 in public hospitals of South Wollo Zone, Northeastern Ethiopia (*n* = 489).

**Variable**	**Category**	**PPE Utilization**		**COR (95%CI)**	**AOR (95%CI)**
		**Poor compliance**	**Good compliance**		
Availability of PPE	No	70	38	1.22 (0.79–1.87)	1.63 (0.93–2.85)
	Yes	263	118	1	1
Feedback for safety	No	281	109	1.42 (0.84–2.43)	2.05 (1.26–3.35)^*^
	Yes	52	47	1	1
Training on COVID-19	No	120	113	4.66 (3.08–7.07)	3.43 (2.01–5.86)^*^
	Yes	213	43	1	1
Perception to infection risk	No	96	93	1.42 (0.95–2.13)	1.98 (1.18–3.33)^*^
	Yes	237	63	1	1
Drinking alcohol	No	275	116	1.64 (1.04–2.58)	0.99 (0.44–2.07)
	Yes	58	40	1	1
Chewing khat	No	297	126	1.96 (1.16–3.33)	1.07 (0.44–2.60)
	Yes	36	30	1	1

*This analysis was adjusted for availability of PPE, feedback for safety, training on COVID-19 prevention, perception to infection risk, drinking alcohol, and chewing chat*.

* *Indicates variables significantly associated with PPE utilization at 95% CI*.

## Discussion

The present study assessed compliance of personal protective equipment (PPE) utilization and hand hygiene practice among 489 healthcare workers toward COVID-19 in public hospital settings in which findings of the present study are very essential to prevent the spread of COVID-19 (25, 26). Because HCWs are the frontline to prevent and control COVID-19, they are at high risk of contracting an infection and can transmit the virus to patients, families, and the community easily (5, 27).

This study result revealed that overall adherence to PPE utilization among healthcare workers was low. The observed utilization of PPE in this study was more frequent than the study findings from Nigeria (28), Gondar, Ethiopia (16), and India (29) in which HCWs who always complied with appropriate use of PPEs ranged from 4.3 to 18.1%. However, it was lower than systematic reviews studies conducted in industrialized countries on compliance with hand hygiene in hospital care: in intensive care units (30–40%), in other departments (50–60%), among physicians (32%), and nurses (48%) (30) and more than 50% in the emergency department (31).

The main reason for low adherence to PPE utilization in this study may due to lack of training about the use and function of PPE utilization for COVID-19 and other disease prevention methods (only half of HCWs reported that they received infection prevention training since the COVID-19 outbreak emerged). Training on infection prevention especially for COVID-19 can enhance compliance of PPE utilization and hand hygiene practice (32) and can reduce the perception of risk (33). Besides, insufficient time, carelessness, discomfort, forgetfulness, lack of habit, and perception of low risk of infection might be other factors for low compliance in PPE utilization.

Hand hygiene is the most essential protective measure to prevent infection, especially SARS-CoV-2. However, the overall compliance of hand hygiene practices among healthcare workers was low. A worldwide systematic review indicated that the overall compliance rate of hand hygiene in hospital care was 40% (30). In this study, lower compliance of hand hygiene practice was reported among doctors (18.9%) than nurses which may be influenced by lack of positive role models and perception of intensified dryness and soreness of hands. It might also be due to the inconvenient placement of the hand rub or sink, no hand rub in the dispenser, or no soap at the sink, being distracted by medical emergencies, low perception of hand hygiene importance to prevent infections, and low safety culture with no feedback for safety.

This study finding also indicated that lack of training on COVID-19 prevention can decrease compliance of PPE utilization among HCWs by more than three-fold (AOR = 3.43). This means that HCWs who had training on COVID-19 prevention were 3.43 times more likely to use the personal protective equipment compared to HCWs who had no previous training on COVID-19 prevention. It is similar to those earlier results found in studies done in Amhara regional state (34), Ethiopia (35), Egypt (36), Tanzania (20), and Italy (33).

Healthcare workers who received frequent feedback on safety practices by institutional management had more than two-fold (AOR = 2.05) higher compliance with PPE utilization. Compliance can be increased through personal or management supervision, instruction, and audit performance by providing feedback for safety (37). This finding is also supported by evidence that HCWs with a good perception of infection risk were nearly two times (AOR = 1.98) more likely to comply with PPE utilization in line with studies done in Addis Ababa, Ethiopia (38), and Italy (33). This indicated that increased perception of infection risk toward COVID-19 might empower HCWs to adhere to PPE utilization against the disease.

## Conclusions

Healthcare workers' compliance on personal protective equipment utilization and hand hygiene practice was inadequate in the public hospitals of South Wollo Zone. The multivariable logistic regression analysis result indicated that feedback for safety, training on COVID-19 prevention, and perception to infection risk were the main predictor variables for compliance of personal protective equipment utilization.

These study results indicate the imperative need for decision-makers to address low compliance on personal protective equipment utilization and hand hygiene practice among HCWs in hospital settings. These findings should inform strategies designed to increase training on COVID-19 prevention and management support for safety to change the behavioral determinants of compliance with the relevant practices. We strongly urge national governments, the private sector, and the general public to pay concerted attention to healthcare worker safety.

## Data Availability Statement

The raw data supporting the conclusions of this article will be made available by the authors, without undue reservation.

## Ethics Statement

The study was conducted in accordance with the Helsinki declaration. The ethical approval letter was obtained from the Institutional Ethical Review Committee of the College of Medicine and Health Sciences of Wollo University with the issue number CMHS/368/13/21. Furthermore, before data collection, written consent was obtained from each healthcare worker. Before data collection, the purpose of the study was explained to all participants and they were assured that their information would not be used for purposes other than scientific research. Participants were informed that participation would be voluntary and that they could withdraw at any time for whatever reason. Confidentiality was maintained by avoiding possible identifiers such as the names of the study participants. Only identification numbers were used as a reference.

## Author Contributions

AK, AA, and MA contributed to the conception and design of the study. AK and AA conducted the investigation. AK, AA, TS, ML, and MA performed data management and analysis. AK, AA, GB, and MA wrote and edited the manuscript. All authors contributed to the article and approved the submitted version.

## Funding

This work was supported by Wollo University (Grant No.: WU/20920/13).

## Conflict of Interest

The authors declare that the research was conducted in the absence of any commercial or financial relationships that could be construed as a potential conflict of interest.

## Publisher's Note

All claims expressed in this article are solely those of the authors and do not necessarily represent those of their affiliated organizations, or those of the publisher, the editors and the reviewers. Any product that may be evaluated in this article, or claim that may be made by its manufacturer, is not guaranteed or endorsed by the publisher.

## Abbreviations

2019-ncov: 2019-novel coronavirus
AOR: adjusted odds ratio
CI: confidence interval
COR: crude odds ratio
COVID-19: coronavirus disease-2019
CSA: Central Statistical Agency
HCWs: healthcare workers
PPE: personal protective equipment
SARS-CoV-2: severe acute respiratory syndrome-coronavirus-2
SPSS: Statistical Package for Social Science
WHO: World Health Organization.
